# FLASH Radiotherapy and Organelle-Targeted Radiosensitization in Glioblastoma: A Conceptual and Translational Review

**DOI:** 10.3390/cancers18111850

**Published:** 2026-06-05

**Authors:** Xielin Tang, Xiaoyi Wang, Kui Xiao, Bingcheng Zhu, Fa Lin, Liangxue Zhou

**Affiliations:** 1 Department of Neurosurgery, The Affiliated Santai Hospital of North Sichuan Medical College, Mianyang 621100, China; 2 Department of Neurosurgery, West China Hospital, West China Medical School, Sichuan University, Chengdu 610041, China; 3 Department of Neurosurgery, NHC Key Laboratory of Nuclear Technology Medical Transformation (Mianyang Central Hospital), School of Medicine, University of Electronic Science and Technology of China, Mianyang 621000, China; 4 Department of Neurosurgery, Beijing Tiantan Hospital, Capital Medical University, Beijing 100070, China

**Keywords:** glioblastoma, FLASH radiotherapy, radioresistance, radiosensitization, endoplasmic reticulum stress, unfolded protein response, tumor microenvironment

## Abstract

Radiotherapy remains a central component of standard treatment for glioblastoma (GBM), yet recurrence is common because GBM radioresistance is reinforced by enhanced DNA damage repair, glioma stem cells (GSCs), hypoxia, extracellular matrix remodeling, and an immunosuppressive tumor microenvironment. FLASH radiotherapy (FLASH-RT), delivered at ultra-high dose rates, has shown reproducible normal-tissue-sparing effects in preclinical models, including the brain. In GBM models, however, available evidence indicates that FLASH-RT generally preserves tumor control at levels comparable to conventional radiotherapy rather than providing clearly superior eradication of hypoxic or stem-like tumor compartments. In parallel, endoplasmic reticulum (ER)-targeted interventions have emerged as a candidate strategy for disturbing tumor proteostasis, modulating unfolded protein response (UPR) signaling, impairing synthesis of repair-associated proteins, and promoting immunogenic cell death. This narrative review summarizes representative mechanisms of GBM radioresistance, appraises the opportunities and limitations of FLASH-RT in intracranial disease, and explains why ER targeting is discussed here as a lead but unproven biological axis for radiosensitization. We further compare ER-directed approaches with mitochondrial-, lysosomal-, and delivery-enabled radiosensitization strategies, and outline the translational variables that would determine clinical testability, including beam modality, blood–brain barrier heterogeneity, pharmacokinetics, treatment sequencing, and biomarker development. In this review, “physical precision” refers primarily to dose-rate-driven ultra-rapid delivery and the possibility of widening the normal-tissue therapeutic window under FLASH conditions, rather than to a universal depth–dose advantage shared by all FLASH platforms. Direct experimental evidence for combining FLASH-RT with ER-targeted therapy in GBM is currently lacking. We therefore present this model as a hypothesis-generating conceptual and translational framework for future preclinical testing rather than as an established therapeutic advance.

## Simple Summary

Glioblastoma remains difficult to treat because resistance to radiotherapy is sustained by efficient DNA repair, glioma stem cells, hypoxia, and an immunosuppressive microenvironment. FLASH radiotherapy delivers radiation within milliseconds at ultra-high dose rates and has shown encouraging normal brain tissue sparing in preclinical studies. However, current evidence does not show that FLASH alone overcomes the intrinsic biological resistance of glioblastoma. This review examines whether organelle-targeted radiosensitization, especially endoplasmic reticulum targeting, could complement FLASH by perturbing proteostasis, weakening repair capacity, and enhancing immunogenic signaling. We discuss this concept as a hypothesis-generating translational framework rather than an established treatment strategy, compare it with other organelle-directed approaches, and outline the preclinical and clinical steps needed to test it rigorously.

## 1. Introduction

Glioblastoma (GBM) is the most common primary malignant brain tumor, accounting for 57% of all gliomas and 48% of primary malignant tumors of the central nervous system [1]. Despite substantial advances in multimodal therapy, including surgery, radiotherapy, and systemic treatment, the overall survival of patients with GBM remains poor, and long-term survival is rare. Radiotherapy remains a central component of postoperative management, but its benefit is constrained by the multifactorial radioresistance of GBM cells [2,3]. FLASH radiation therapy (FLASH-RT) has emerged as an important radiation-delivery concept because it can preserve normal brain tissue in several preclinical settings while maintaining tumor control comparable to conventional radiotherapy (CONV-RT) [4,5]. However, current GBM evidence does not demonstrate that FLASH-RT alone eliminates hypoxic or stem-like resistant compartments more effectively than CONV-RT. In parallel, GBM cells show functional dependence on subcellular stress-response systems, including endoplasmic reticulum (ER) proteostasis and the unfolded protein response (UPR), which may be therapeutically exploitable when these adaptive pathways are pushed beyond their compensatory capacity [6,7,8,9]. We therefore review FLASH-RT and organelle-targeted radiosensitization as intersecting but still experimentally unvalidated concepts. The purpose of this review is to define a hypothesis-generating framework, clarify its evidence boundaries, and identify the preclinical requirements that must be met before FLASH-RT plus ER-targeted therapy can be considered for clinical testing.

## 2. Representative Mechanisms of Radioresistance in Glioblastoma

The following subsections summarize representative mechanisms most relevant to the later discussion of radiosensitization rather than attempting an exhaustive catalogue of all contributors to GBM treatment failure.

### 2.1. Aberrant Activation of DNA Damage Repair Pathways

In GBM cells, particularly GSCs, aberrant activation or overexpression of key components of DNA double-strand break (DSB) repair pathways represents a central mechanism of radioresistance. Non-homologous end joining (NHEJ) and homologous recombination (HR) are the two principal pathways responsible for repairing the lethal DNA damage induced by radiotherapy. Studies have shown that GSCs enhance NHEJ activity through upregulation of apoptosis-antagonizing transcription factor (AATF). Mechanistically, AATF interacts with the core NHEJ subunit XRCC4 and prevents its degradation through the ubiquitin–proteasome pathway, thereby sustaining efficient DNA repair and promoting therapeutic resistance [10]. Similarly, increased activity of DNA-dependent protein kinase catalytic subunit (DNA-PKcs) and ataxia–telangiectasia mutated (ATM), which are key regulators of the NHEJ and HR pathways, has been closely associated with radioresistance [11]. Upregulation of specificity protein 1 (Sp1) can activate the DNA-PKcs promoter, thereby increasing its expression and activity, promoting DSB repair, and ultimately contributing to radioresistance in GBM cells [11]. Likewise, the UBA1 inhibitor TAK-243 has been shown to enhance the radiosensitivity of GBM cells by impairing DNA damage repair, particularly through inhibition of recruitment of the downstream effector 53BP1 [12]. Collectively, these findings suggest that targeting key DNA repair proteins such as DNA-PKcs and ATM may represent an important strategy for overcoming GBM radioresistance.

Epigenetic regulation plays a crucial role in the expression of DNA repair-related genes. Demethylation of the O6-methylguanine-DNA methyltransferase (MGMT) promoter, leading to its high expression, is a classic mechanism that weakens the efficacy of the alkylating agent temozolomide (TMZ) and indirectly affects radiotherapy response [13]. Beyond DNA methylation, post-translational modifications are also critical. For example, interferon-induced guanylate-binding protein 3 induces the expression of p62, NRF2, and MGMT by stabilizing STING protein levels, thereby leading to TMZ resistance [14]. These regulatory networks at the epigenetic and post-translational levels enable GBM cells to flexibly adjust their DNA repair capacity, forming a complex basis for therapy resistance. Inhibitors targeting the aforementioned DNA damage repair pathways have shown significant radiosensitization potential in preclinical studies. For instance, using nanoparticles to deliver small interfering RNAs targeting ATM and DNA-PKcs can effectively silence target protein expression, leading to persistent DNA damage markers (γ-H2AX foci) and enhancing GBM radiosensitivity both in vitro and in vivo [15]. However, due to redundancy and compensatory mechanisms between pathways, targeting a single repair pathway often yields limited efficacy. Studies indicate that GBM cells can simultaneously activate HR and NHEJ pathways to promote therapy resistance [16]. Therefore, combination inhibition strategies appear more effective. For example, simultaneous inhibition of ATM and the cystine/glutamate antiporter can concurrently disrupt DSB repair and redox homeostasis, thereby significantly enhancing GBM radiosensitivity and inhibiting tumor growth both in vitro and in vivo [17]. These studies emphasize that developing multi-target combination strategies or targeting core regulatory nodes of repair pathways can help overcome GBM radioresistance.

### 2.2. The Key Role of GSCs

The failure of GBM treatment and subsequent recurrence are largely attributed to the presence of GSCs. GSCs represent a subpopulation within GBM characterized by self-renewal capacity, multilineage differentiation potential, and high tumorigenicity, and are widely regarded as the “seed” cells that drive tumor growth, recurrence, and therapeutic resistance [18,19]. Numerous studies have shown that GSCs display pronounced resistance to CONV-RT and chemotherapy, and that their intrinsic biological properties form a major basis of radioresistance [20,21]. For example, the ability of patient-derived GSCs to form neurospheres in vitro is closely associated with the maintenance of stemness and tumorigenic potential. Targeting key molecules in GSCs, such as GDF15, GPM6A, or members of the PEA3 transcription factor family (ETV1, ETV4, and ETV5), can significantly impair their self-renewal capacity and enhance their radiosensitivity [18,19,22]. These findings underscore the central role of GSCs in GBM pathophysiology and highlight the importance of understanding their resistance mechanisms for optimizing radiotherapy strategies.

The radioresistance of GSCs arises from their distinct cellular state. First, GSCs often reside in a relatively quiescent state, which enables them to evade CONV-RT that primarily targets rapidly proliferating cells [20]. Second, they express high levels of anti-apoptotic proteins, such as members of the Bcl-2 family, and exhibit robust DNA damage checkpoint activation and highly efficient DNA repair capacity, allowing them to rapidly repair radiation-induced DSBs, preserve genomic stability, and survive [23,24]. For example, replication protein A (RPA) is highly expressed in GSCs, and its depletion impairs GSC survival and self-renewal while sensitizing these cells to radiation [25]. In addition, GSCs undergo distinct metabolic reprogramming, including a preferential reliance on oxidative phosphorylation (OXPHOS) to meet their energy demands [26]. This metabolic phenotype is closely linked to mitochondrial function, which plays a crucial role in maintaining GSC stemness and therapy resistance [27]. Studies have shown that agents targeting mitochondrial protein translation, such as doxycycline, can induce mitochondrial dysfunction and cell death in GSCs while exerting less pronounced effects on differentiated glioma cells, further highlighting the importance of metabolic pathways in GSC-associated resistance [27].

GSCs do not exist in isolation; rather, their survival and function are highly dependent on the TME. They are preferentially enriched in hypoxic regions and perivascular niches, where local conditions further reinforce stemness and therapeutic resistance through multiple signaling pathways [28]. Under hypoxic conditions, members of the hypoxia-inducible factor (HIF) family, including HIF-1α and HIF-2α, are activated and regulate a broad range of genes involved in cell survival, metabolism, and stemness [29]. Studies using temperature-responsive PNJ scaffolds to recapitulate the microenvironment have shown that HIF-2α expression is increased in scaffold-cultured GSCs and is associated with enhanced self-renewal and radioresistance [29]. In addition, microenvironmental cues such as neuronal activity can induce a proneural-to-mesenchymal transition (PMT) in GSCs through the release of exosomes enriched in miR-184-3p, thereby promoting tumor progression and radioresistance [30]. Intercellular interactions and signaling molecules within the perivascular niche, such as integrin α6, are also critical, either supporting stem cell properties or promoting radioresistance in GSCs across different molecular subtypes [31]. Accordingly, targeting these microenvironment-dependent signaling pathways, for example by using the antiepileptic drug levetiracetam to reduce the production of neuron-derived exosomes, may represent a promising strategy for overcoming GSC-associated therapeutic resistance [30].

### 2.3. Synergistic Promotion by the TME

The TME of GBM is a highly complex and dynamic ecosystem. Its defining features, including severe hypoxia, acidosis, aberrant angiogenesis, and a profoundly immunosuppressive state, collectively provide a critical foundation for tumor progression and therapeutic resistance. Hypoxia is one of the best-established drivers of radioresistance in the TME, as it directly attenuates the cytotoxic effects of radiation by limiting the generation of radiation-induced free radicals [32]. More importantly, stabilization of HIF-1α represents a central mechanism underlying hypoxia-mediated radioresistance. Studies have shown that the metabolite L-2-hydroxyglutarate (L-2-HG) can increase HIF-1α protein levels and upregulate its downstream target genes, thereby promoting radioresistance, proliferation, and migration in GBM cells [33]. This metabolically driven activation of HIF-1α signaling highlights the close link between tumor metabolic adaptation and resistance to radiotherapy. Acidosis within the TME is another important contributor to radioresistance and primarily arises from the Warburg effect, in which tumor cells preferentially rely on aerobic glycolysis [34]. Studies have demonstrated that an acidic microenvironment can activate the proton-sensing G protein-coupled receptor GPR68, which promotes tumor cell survival through inhibition of ATF4. Conversely, inhibition of GPR68 can induce ferroptosis and enhance the sensitivity of various cancer cells to ionizing radiation, suggesting that GPR68 is a key mediator of acidosis-associated radioresistance [34,35].

In the context of immunosuppression, tumor-associated macrophages and microglia constitute the predominant immune cell population within the GBM microenvironment, and their phenotypic polarization has a major impact on therapeutic outcomes. Accumulating evidence indicates that radioresistant GBM microenvironments are enriched in M2-like macrophages, and that the M1/M2 macrophage ratio is negatively correlated with the radiosensitivity index, indicating that a higher proportion of M2 macrophages is associated with greater tumor radioresistance [36]. These M2-polarized macrophages establish a microenvironment favorable to tumor growth and treatment resistance through the secretion of immunosuppressive cytokines such as TGF-β and IL-10 [37]. For example, cyclooxygenase-2 can drive tumor-associated macrophages toward an M2-like phenotype through its product prostaglandin E2, thereby supporting the self-renewal of GSCs and reinforcing both immunosuppression and therapeutic escape [38]. In turn, GSCs can upregulate CD47 expression through AMPK-mediated epigenetic regulation following radiotherapy, thereby inhibiting macrophage phagocytosis and promoting M2-like polarization, which further reshapes the immunosuppressive TME in favor of treatment evasion [39]. In addition, triggering receptor expressed on myeloid cells-2 (TREM2) and high mobility group box 1 (HMGB1) have been reported to form a positive feedback loop that activates the TLR4/Akt signaling pathway, thereby accelerating GBM radioresistance and immune escape [40].

**Figure 1:**
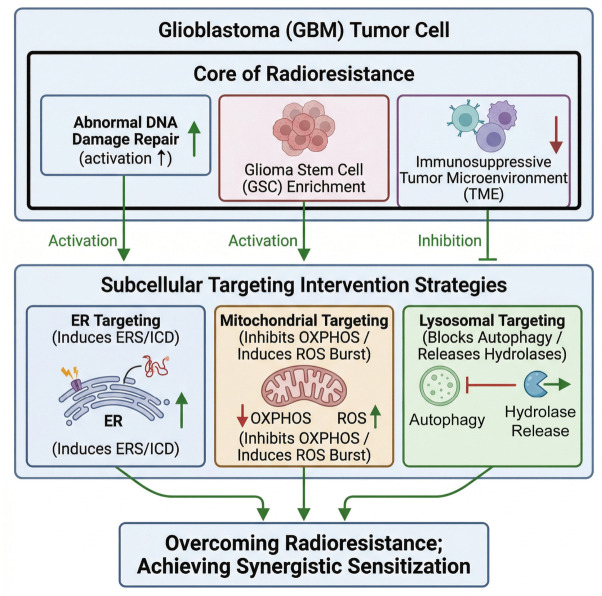
Core mechanisms of glioblastoma radioresistance and subcellular organelle-targeted intervention strategies. GBM radioresistance arises from three interrelated biological layers: aberrant activation of DNA damage repair pathways, enrichment of GSCs, and establishment of an immunosuppressive TME. The upper panel highlights key features of these mechanisms, including upregulation of NHEJ and HR pathway proteins, maintenance of GSC stemness and therapeutic resistance, M2-type macrophage polarization, expansion of myeloid-derived suppressor cells, and CD8+ T-cell exhaustion. The middle panel depicts subcellular organelle-targeting strategies that disrupt tumor cell homeostasis through ER targeting, mitochondrial targeting, and lysosomal targeting. The lower panel illustrates the potential synergistic effects of these strategies in enhancing radiosensitivity and overcoming therapeutic resistance in GBM.

Abnormal extracellular matrix (ECM) remodeling and elevated interstitial fluid pressure not only form physical barriers that impede drug delivery, but also actively promote tumor cell survival, invasion, and radioresistance through integrin-mediated and related signaling pathways. Studies have shown that the expression signature of ECM receptor pathway genes is significantly associated with radiotherapy response and prognosis in glioma patients [41]. In particular, simultaneous inhibition of discoidin domain receptor 1 (DDR1) and integrins αVβ3/αVβ5 most effectively reduces the clonogenic potential of GBM cells, enhances radiosensitivity, and impairs DSB repair [42]. These findings underscore the crucial role of ECM-receptor interactions in the DNA damage response. Epigenetic regulation also contributes to ECM-mediated radioresistance. Following radiotherapy, bromodomain-containing protein 4 (BRD4)-dependent super-enhancers drive the expression of type I collagen (COL1A1), thereby promoting ECM remodeling and radioresistance; conversely, BRD4 inhibition can enhance radiosensitivity by downregulating COL1A1 [43]. Metabolic reprogramming is likewise intertwined with ECM remodeling. In radioresistant GBM cells, upregulation of the mitochondrial glutamate transporter SLC25A22 leads to cytoplasmic glutamate accumulation. This, on the one hand, enhances glutathione synthesis to counteract radiation-induced reactive oxygen species and, on the other hand, promotes proline synthesis, thereby driving ECM remodeling and ultimately conferring a more invasive and radioresistant phenotype [44]. Biomimetic studies using three-dimensional engineered hydrogel models have further confirmed that ECM mechanical cues, such as matrix stiffness, together with oxygen availability, jointly regulate cellular responses to radiation. Softer matrices permit greater DNA damage, whereas hyaluronic acid fragments modulate rapid metabolic responses to radiation under hypoxic conditions [45]. Taken together, these findings emphasize the central role of the TME in shaping GBM radioresistance and highlight the importance of synergistic strategies that simultaneously target hypoxia, immunosuppression, and ECM remodeling in order to overcome therapeutic resistance. These interrelated resistance mechanisms and the corresponding organelle-targeted intervention concepts are summarized in Figure 1.

## 3. FLASH-RT: Principles, Advantages, and Challenges in GBM Treatment

### 3.1. Physical and Biological Characteristics of FLASH-RT

FLASH-RT is generally defined as a form of radiotherapy delivered at ultra-high dose rates (typically >40 Gy/s) over an extremely short time frame, usually within milliseconds to seconds, in sharp contrast to conventional dose-rate radiotherapy, which is generally delivered at dose rates of approximately 0.01–0.1 Gy/s [46]. This technological approach seeks to complete radiation delivery within a very brief irradiation period, thereby potentially reducing complications associated with prolonged treatment times, such as organ motion [47]. From a physical standpoint, the implementation of FLASH-RT depends on advanced accelerator technologies capable of generating and precisely controlling ultra-high-dose-rate beams, including modified linear accelerators, proton or carbon-ion systems, and laser-plasma accelerators [48]. Its defining biological feature is the so-called “FLASH effect”, whereby equivalent physical doses can substantially reduce both acute and late toxicity in normal tissues while preserving, or potentially enhancing, antitumor efficacy [49]. The identification of this effect has introduced a promising avenue for improving the therapeutic ratio of radiotherapy, that is, the balance between tumor control and normal tissue injury [50].

The mechanisms underlying the FLASH effect remain incompletely understood, and several non-mutually exclusive hypotheses have been proposed. One of the most widely discussed is differential oxygen depletion kinetics, according to which ultra-high-dose-rate irradiation may induce rapid and transient oxygen depletion within irradiated tissues, leading to a brief hypoxic state in normal tissues and thereby reducing their radiosensitivity [51]. However, computational modeling and experimental measurements suggest that the extent of oxygen depletion induced by FLASH irradiation at clinically relevant doses may be insufficient to account fully for the observed degree of radioprotection, leaving this hypothesis open to debate [52]. Another explanation centers on free-radical chemistry. Under ultra-high dose-rate conditions, radiation-induced reactive oxygen species (ROS) and other free radicals may accumulate at very high local concentrations over a short time interval, increasing the likelihood of radical-radical recombination and thereby reducing the number of radicals available to damage critical biomolecules such as DNA [53]. In addition, immune modulation and activation of specific signaling pathways have also been proposed as contributing mechanisms. For example, emerging evidence suggests that FLASH-RT may render the TME more immunogenic, thereby creating opportunities for combination with immunotherapy [46]. At the same time, FLASH-RT may differentially influence metabolic responses in normal and tumor tissues by affecting mitochondrial function, iron metabolism, and lipid peroxidation, which may further contribute to its tissue-sparing effects [54].

A substantial body of preclinical evidence has demonstrated normal tissue protection by FLASH-RT across multiple animal models, including the brain, lung, and skin [55]. For example, in mouse models, FLASH proton irradiation reduced normal tissue injury, such as acute skin wet desquamation and late radiation-induced fibrosis, compared with conventional dose-rate proton pencil beam scanning, while maintaining equivalent tumor control [56]. In rodent models of whole-brain irradiation, FLASH-RT has also shown the potential to preserve neurocognitive function [55]. Nevertheless, important challenges remain, including incomplete understanding of its molecular basis, lack of standardized dosimetric and biological parameters, and platform-specific delivery constraints. Limited penetration is a major limitation for many current electron-FLASH systems, but it should not be generalized to all FLASH modalities. Transmission proton FLASH concepts for deep-seated targets often use proton energies of approximately 200–250 MeV, whereas candidate very-high-energy electron (VHEE) platforms span roughly 50–250 MeV; hybrid UHDR/conventional strategies and other particle or photon approaches are also under development for intracranial targets [57,58,59].

### 3.2. Application and Limitations of FLASH-RT in GBM Models

In preclinical models of GBM, FLASH-RT has demonstrated substantial advantages in protecting normal brain tissue while maintaining effective tumor control. Multiple studies have shown that, compared with CONV-RT, FLASH-RT more effectively preserves cognitive function, particularly in vulnerable structures such as the hippocampus. For example, in mouse models bearing intracranial GBM, single high-dose or hypofractionated FLASH-RT was as effective as CONV-RT in delaying tumor growth; however, only FLASH-RT significantly reduced radiation-induced deficits in learning and memory in tumor-bearing animals [60]. This neuroprotective effect has also been observed in juvenile mouse models, in which FLASH-RT protected both developing and mature neurons, reduced microglial proliferation, and prevented cognitive impairment across multiple behavioral tests, adverse effects that were commonly observed in the CONV-RT group [61]. Evidence from proton FLASH-RT studies further supports these findings. In rat glioma models, FLASH proton therapy avoided the memory impairment induced by conventional high-dose proton therapy while eliciting a similar degree of tumor-infiltrating lymphocyte recruitment [62]. In addition, mechanistic studies of normal brain protection have shown that FLASH-RT can preserve blood–brain barrier integrity, reduce vascular dilation, and prevent the loss of tight junction proteins such as occludin and claudin-5, all of which are pathological changes commonly observed after CONV-RT [63]. A multi-institutional study further confirmed the reproducibility of the FLASH effect: following whole-brain irradiation of mice using different electron beam platforms, FLASH-RT preserved novel object recognition and overall behavioral performance, whereas CONV-RT resulted in cognitive decline [64]. Collectively, these findings indicate that FLASH-RT can achieve a favorable separation between tumor control and normal brain tissue injury in GBM models.

Despite these protective advantages, the available evidence suggests that the direct tumoricidal efficacy of FLASH-RT against GBM does not consistently exceed that of CONV-RT. Multiple preclinical studies conducted in fully immunocompetent animal models have shown that FLASH-RT and CONV-RT are similarly effective in suppressing tumor growth and prolonging survival. For example, in intracranial NS1 GBM models in Fischer 344 rats, both single-dose 20 Gy and 25 Gy FLASH-RT and CONV-RT significantly prolonged survival and exhibited comparable dose-response relationships [4]. Another study similarly demonstrated that, in both subcutaneous and intracranial GBM models, FLASH and CONV achieved equivalent efficacy under fractionation schedules of 8 Gy × 2 or 12.5 Gy × 2, and animals cured by either approach developed long-term antitumor immunity [5]. Likewise, studies using proton beams in patient-derived xenograft (PDX) models of intracranial GBM found no difference in tumor control between FLASH and conventional proton radiotherapy [65]. More detailed mechanistic analyses have also shown no significant differences between FLASH-RT and CONV-RT in the induction of DSBs or chromosomal translocations [66]. Notably, the potential advantage of FLASH-RT in eradicating hypoxic cells, which are abundant in GBM, remains uncertain and may in fact be limited. One hypothesis proposes that ultra-fast dose delivery may attenuate the oxygen fixation effect, such that insufficient radiochemical oxygen depletion (ROD) could reduce the killing efficiency of hypoxic cells. In spheroid tumor models, FLASH-RT and CONV-RT showed no difference in sterilizing efficacy against U87 GBM spheroids under normoxic conditions; however, under hypoxic conditions, FLASH-RT required significantly higher doses to achieve the same sterilizing effect [67]. An in vivo dosimetric study further reported that although ultra-high-dose-rate (UHDR) irradiation induced measurable changes in tissue oxygenation at clinically relevant doses and dose rates, these changes did not support ROD-induced radioresistance as the principal explanation for the normal tissue-sparing effect of FLASH [68]. Taken together, these findings suggest that FLASH-RT may provide little, if any, advantage in overcoming hypoxia-driven radioresistance in GBM and, under certain conditions, may even be less effective. This limitation represents a major challenge for the application of FLASH-RT in GBM treatment.

From a technical implementation perspective, the application of FLASH-RT to intracranial tumors still faces substantial challenges. Equipment capable of delivering UHDR irradiation with the precision required for complex intracranial targets remains under active development and optimization. Conventional electron FLASH, because of its limited penetration depth, is not well suited for deep-seated tumors. Consequently, alternative particle beams and hybrid delivery approaches are being actively explored. For example, transmission proton (TP) beams and very high-energy electron (VHEE) beams have been proposed as promising options for extending the FLASH effect to deep intracranial targets [57]. Treatment planning studies have shown that VHEE-based three-dimensional conformal radiotherapy (3D-CRT) for brain tumors such as GBM can achieve dosimetric plan quality comparable to that of standard intensity-modulated photon radiotherapy (IMRT) while using only a limited number of beams (e.g., 3–7 beams) [58]. In addition, hybrid treatment strategies combining UHDR electrons with conventional dose-rate photons have been proposed as a dosimetrically feasible solution for conformal UHDR treatment of deep-seated targets, including glioblastoma, thereby helping to address some of the major technical limitations [59]. Another emerging approach involves the combination of spatially fractionated microbeam GRID therapy with VHEE-based FLASH, with preliminary studies suggesting feasibility in pediatric GBM models [69]. Beyond beam delivery systems, precise dosimetry and real-time treatment verification remain equally demanding requirements and represent major barriers to the clinical translation of FLASH-RT. One study demonstrated dosimetric reproducibility across two institutions using different electron FLASH devices, standardized phantoms, and dosimeters, and further showed that the normal brain-sparing effects of FLASH were reproducible across institutions and beam platforms [64]. Another investigation compared the dosimetric and biological performance of electron and proton FLASH beams and, despite marked differences in temporal microstructure, established dosimetric benchmarks and confirmed that both modalities could achieve normal brain protection while maintaining tumor control [70]. These technological advances and standardization efforts will be essential for the safe and effective clinical application of FLASH-RT in GBM.

Taken together, these studies suggest that the principal contribution of FLASH-RT in GBM is improvement of the safety profile of radiotherapy through normal brain sparing, rather than direct and reproducible reversal of intrinsic tumor radioresistance. The broader concept of combining radiotherapy with organelle-targeted radiosensitization is not unique to FLASH-RT and could, in principle, be adapted to other advanced platforms such as proton or carbon-ion therapy. In this review, FLASH-RT is emphasized because its most reproducible preclinical signal in the brain is normal-tissue sparing, which may be particularly relevant when adding pharmacological or nanomedicine-mediated stress to cranial irradiation. Thus, the following sections should be read as a conceptual framework for future testing rather than as evidence that FLASH-RT is indispensable or already synergistic in GBM.

Clinically, the potential value of normal brain sparing should be interpreted as an enabling rather than directly tumoricidal advantage. If confirmed in humans, it could permit safer hypofractionation, focal dose escalation, re-irradiation of recurrent disease, or combination with radiosensitizers by reducing the risk of neurocognitive, vascular, and BBB-related toxicity. However, direct clinical evidence that FLASH-RT improves survival or functional outcomes in patients with GBM is not yet available, and the current rationale is therefore inferred primarily from animal data, treatment-planning studies, and early translational development.

## 4. Endoplasmic Reticulum Stress as a Candidate Radiosensitization Axis in GBM

### 4.1. ERS and UPR Dependency as a Candidate Vulnerability in GBM

Rapid proliferation and high biosynthetic demand impose substantial pressure on ER proteostasis in GBM cells, but increased ER workload alone does not automatically establish therapeutic vulnerability. The more relevant rationale is that GBM cells can depend on adaptive UPR signaling, including IRE1, PERK, ATF6, and GRP78/BiP, to sustain tumor initiation, progression, angiogenesis, stem-cell maintenance, and interactions with the tumor microenvironment [24]. It is this functional dependence on stress-adaptation pathways, rather than ER activity per se, that supports investigation of ER-targeted therapy as a candidate radiosensitization axis in GBM.

Disruption of ER homeostasis activates the UPR, an adaptive signaling network that initially functions to restore proteostasis. When ERS is excessive or prolonged and homeostasis cannot be re-established, the UPR can shift from a cytoprotective response to a pro-death program, ultimately promoting apoptosis and, in some contexts, immunogenic cell death (ICD) [3]. Deliberately pushing ERS beyond the adaptive threshold may therefore weaken GBM survival programs, but this concept remains context-dependent and requires direct validation in clinically relevant GBM models.

### 4.2. Intervention Strategies: Pharmacological Approaches Targeting the ER

Pharmacological induction of ERS represents one of the principal approaches for targeting the ER in GBM. In most cases, this strategy involves disruption of protein homeostasis, leading to the accumulation of misfolded or unfolded proteins within the ER lumen, activation of intense ERS and UPR signaling, and ultimately induction of cell death [28].

Proteasome inhibitors. Carfilzomib (CFZ), for example, induces ERS through inhibition of the ubiquitin–proteasome system. When co-delivered with calcium peroxide (CaO_2_) nanoparticles, CFZ has shown synergistic antitumor effects and superior efficacy compared with monotherapy in preclinical models [28].

Natural products and molecular chaperone inhibitors. Betulinic acid (BA) can induce ERS-mediated cell death by suppressing Sp1 and activating the PERK/CHOP apoptotic pathway, and this effect has also been observed in TMZ-resistant GBM cells [28]. In addition, the GRP78 inhibitor HA15 has been shown to synergize with other agents to induce apoptosis through activation of the PERK/ATF4 and IRE1α/XBP1 signaling axes, resulting in marked inhibition of intracranial GBM growth in preclinical models [28].

### 4.3. Potential Mechanisms for Reversing Radioresistance

Targeted induction of ERS may counteract several core features of GBM radioresistance by disrupting tumor cell proteostasis from multiple angles [34].

#### 4.3.1. Interfering with DNA Damage Repair Capacity

The strong DNA damage response (DDR) capacity of GBM is a major contributor to its radioresistance. Increasing evidence suggests that functional crosstalk exists between the UPR, activated by ERS, and DDR pathways [34]. In particular, activation of the PERK/eIF2α signaling axis leads to global suppression of protein translation, thereby reducing the synthesis of key DNA repair proteins, including DNA-PKcs, ATM, and BRCA1. As a result, the repair of radiation-induced DSBs may be impaired [34]. Preclinical studies have shown that PERK-targeting inhibitors not only reverse therapy-induced senescence but also significantly reduce the expression of DNA repair-related proteins, thereby enhancing the radiosensitivity of GBM cells [34].

#### 4.3.2. Eliminating GSCs and Remodeling the Immune Microenvironment

GBM radioresistance is also closely linked to the enrichment of GSCs and the establishment of an immunosuppressive TME [15]. ER-targeted strategies may simultaneously address both of these resistance-associated features through the induction of ICD [3]. When ERS is persistent and unresolved, it triggers apoptosis and releases damage-associated molecular patterns (DAMPs), a process known as ICD [71,72]. ICD can promote antitumor immune responses, help reverse the immunosuppressive TME, and may also contribute to the elimination of treatment-resistant GSC subpopulations [15].

Taken together, GBM radioresistance represents a complex network shaped by DNA repair capacity, stem cell-associated traits, and microenvironmental adaptation [73,74]. ER-targeted strategies may affect several of these determinants by perturbing proteostasis, limiting repair protein synthesis, and promoting immunogenic signaling. However, the evidence supporting this rationale is largely derived from ER stress biology and conventional radiotherapy radiosensitization studies rather than direct GBM experiments combining ER targeting with FLASH-RT. ER targeting should therefore be viewed as a biologically plausible but currently unvalidated component of combination therapy.

Despite this rationale, ER-targeted radiosensitization remains constrained by several unresolved limitations, including heterogeneous BBB access, uncertain subcellular delivery efficiency, potential neurotoxicity in normal brain tissue, adaptive pro-survival UPR signaling, and the absence of direct FLASH-combination data in GBM. These limitations should be considered prerequisites for experimental design rather than minor implementation details.

### 4.4. Potential Safety Risks of Targeting ERS

#### 4.4.1. Neurotoxicity Risk to Normal Brain Tissue

The ER is a central organelle for maintaining protein homeostasis. In normal brain tissue, neurons and astrocytes exhibit high metabolic activity and complex protein synthesis requirements, making their function highly dependent on intact ER homeostasis [4]. Systemic administration of ERS inducers may therefore interfere non-selectively with ER function in normal neural cells. For example, benzo[a]pyrene exposure has been shown to induce ERS in human astroglioma cells, with upregulation of stress markers such as BiP, PERK, and IRE1 [13]. When ERS becomes excessive or the UPR fails to restore homeostasis, apoptosis may also be triggered in nonmalignant neural cells. Consistent with this, studies in models of ischemic brain injury have shown that oxygen-glucose deprivation can induce ERS in astrocytes, resulting in increased apoptosis and autophagy [14]. Preclinical studies have further reported that certain environmental toxicants, such as perfluorooctanoic acid, can induce astrocyte activation and neuroinflammation through the ERS-autophagy axis, thereby recapitulating features of potential drug-related neurotoxicity. Although GBM cells may be more vulnerable to further ERS because of their high proliferative rate and abnormal protein metabolic burden, this differential sensitivity may still offer only a narrow therapeutic window [7]. Without selective delivery strategies, ERS-inducing agents may affect tumor cells and normal neural cells simultaneously, potentially causing irreversible neurological injury, including cognitive dysfunction, neuroinflammation, and neuronal loss. Such off-target toxicity could ultimately limit both the maximum tolerated dose and the therapeutic efficacy of these agents.

#### 4.4.2. Risk of UPR Activation Promoting Tumor Adaptive Survival and Acquired Resistance

The primary physiological role of the UPR is to enable cells to adapt and survive under stress conditions. Under sublethal ERS, the UPR restores homeostasis by upregulating molecular chaperones such as GRP78/BiP to enhance protein-folding capacity, transiently suppressing global protein translation to reduce ER burden, and activating adaptive pathways such as autophagy [4]. If the intensity or duration of targeted therapy is insufficient to exceed the compensatory threshold of tumor cells, such treatment may inadvertently promote adaptation by activating these pro-survival pathways. For example, glioma cells can exploit UPR sensors, including PERK, IRE1, and ATF6, to maintain a chemoresistant state [4]. Sustained low-level UPR activation has also been associated with increased tumor aggressiveness. One study established an ERS-driven, senescence-associated tumor aggressiveness gene signature and identified the UPR as a key senescence-related mechanism promoting glioma invasiveness [15]. Such adaptive responses may ultimately render tumor cells more resistant to subsequent therapy. Thus, incomplete or suboptimal ERS induction carries the risk of promoting malignant progression rather than suppressing it. For instance, FKBP9 expression in glioma cells not only enhances malignant phenotypes through modulation of the IRE1α-XBP1 pathway but also confers resistance to ERS-inducing agents [75]. Therefore, treatment regimens must be carefully optimized to avoid unintentionally promoting tumor survival and acquired resistance through inappropriate dosing or timing.

#### 4.4.3. Core Strategies for Achieving Selective Killing and Risk Mitigation

(1) Tumor-Targeted Delivery Systems Based on Nanotechnology

Nanotechnology-based targeted delivery systems represent a key strategy for achieving precise delivery of ERS inducers in GBM and improving therapeutic selectivity. These platforms exploit distinctive pathophysiological features of GBM, such as localized blood–brain barrier (BBB) disruption and the enhanced permeability and retention (EPR) effect, allowing nanoparticles, including liposomes and polymeric micelles, to accumulate passively within the tumor region [16]. To further improve targeting efficiency, nanocarrier surfaces can be functionalized with specific ligands, such as anti-CD44 antibodies or transferrin receptor-binding moieties, to actively recognize markers overexpressed on GBM cells or tumor-associated vasculature. This approach can increase intratumoral drug accumulation while reducing toxicity to normal brain tissue and the systemic circulation [17]. In addition, the development of smart responsive nanocarriers provides another important means of improving selectivity. These systems can undergo structural changes or degradation in response to tumor-specific stimuli, such as mild acidity, high reducing potential, or enzyme overexpression, thereby enabling site-specific release of encapsulated ERS inducers, including proteasome inhibitors or tunicamycin analogs [3]. Such spatiotemporally controlled delivery strategies may not only improve drug bioavailability but also minimize off-target effects on normal cells, thereby providing an important safeguard for balancing the safety and efficacy of ER-targeted therapy [4].

(2) Synergistic Sensitization Strategies Exploiting TME Characteristics

Designing synergistic sensitization strategies that take advantage of the distinctive characteristics of the GBM TME is another effective approach for enhancing the cytotoxic effects of ERS while reducing injury to normal cells. The GBM microenvironment is often characterized by high oxidative stress, which may constitute a therapeutic vulnerability. Combining ERS inducers with agents capable of selectively generating ROS at the tumor site, such as low-dose calcium peroxide nanoparticles, may further deplete limited antioxidant reserves, including glutathione, within tumor cells and thereby generate a strong synergistic killing effect [18]. This synergy can intensify oxidative folding stress within the ER, leading to rapid collapse of ER function in tumor cells, while exerting less impact on normal cells with more intact redox homeostasis [19]. In parallel, targeting metabolic vulnerabilities associated with GBM reprogramming also represents an important strategy. GBM cells display distinct metabolic features, including enhanced glycolysis and altered glutamine utilization. Combining ERS-targeted agents with drugs that disrupt energy metabolism or glutamine metabolism may reduce the energy and substrate availability required for tumor cells to cope with ERS, thereby weakening their compensatory UPR capacity [20]. For example, inhibition of fatty acid synthase has been shown to induce oxidative stress and ERS while simultaneously suppressing tumor invasion and stem-like phenotypes [18]. Such metabolic intervention strategies may shift sublethal ERS toward irreversible, apoptosis-inducing ERS, thereby promoting more selective elimination of tumor cells [5].

(3) Spatiotemporal Synergistic Integration with Advanced Radiotherapy Techniques

To distinguish established findings from unvalidated hypotheses, Table 1 summarizes the current evidence boundaries for FLASH-RT-based organelle-targeted radiosensitization in GBM and indicates whether each claim is supported by direct GBM or FLASH-specific evidence.

Spatiotemporal coordination between ERS-targeted therapy and advanced radiotherapy is attractive, but it should be framed as a staged translational hypothesis rather than an established treatment strategy. FLASH-RT may expand the normal brain safety window, whereas nanocarrier- or ligand-enabled ERS induction may help concentrate biological stress in residual tumor compartments. The feasibility of this concept will depend on biodistribution, pharmacokinetics, BBB penetration, intracellular trafficking, and whether the selected agent can reach infiltrative margins without unacceptable neurotoxicity. Thus, any proposed integration of FLASH-RT with ER-targeted therapy requires direct orthotopic GBM testing before clinical translation.

## 5. Conceptual Basis for Combining FLASH-RT with ER-Targeted Therapy

### 5.1. Dose-Rate-Driven Normal-Tissue Sparing and Biological Radiosensitization

In this review, physical precision refers primarily to dose-rate-driven ultra-rapid delivery and the possibility of widening the normal-tissue therapeutic window under FLASH conditions. It does not imply a universal depth–dose advantage shared by all FLASH platforms; depth–dose selectivity depends on beam modality, energy, geometry, and treatment planning. Accordingly, the relevance of FLASH-RT to GBM combination therapy lies mainly in its potential to reduce normal brain injury while maintaining tumor control, thereby creating a safer context in which additional radiosensitizing interventions could be tested.

Thus, normal brain sparing is relevant to GBM combination therapy not because it independently eradicates resistant tumor cells, but because it may increase the tolerability of intensified or combined strategies. In practical terms, this benefit could support safer focal boost approaches, treatment of infiltrative margins, re-irradiation in recurrent disease, or the addition of radiosensitizers, provided that UHDR dosimetry and drug-related toxicities are rigorously controlled. These potential clinical advantages remain hypothetical until they are demonstrated in human GBM settings.

Residual GBM disease remains biologically heterogeneous, with hypoxic regions, stem-like niches, and infiltrative margins contributing to recurrence [76]. ER-targeted therapies, photodynamic or sonodynamic approaches, radiodynamic nanoplatforms, and other organelle-directed strategies may provide ways to intensify treatment against these residual compartments. At present, however, most evidence supporting these approaches comes from conventional irradiation or non-FLASH experimental systems. Their relevance to FLASH-RT therefore remains a testable hypothesis rather than a demonstrated therapeutic interaction.

**Figure 2:**
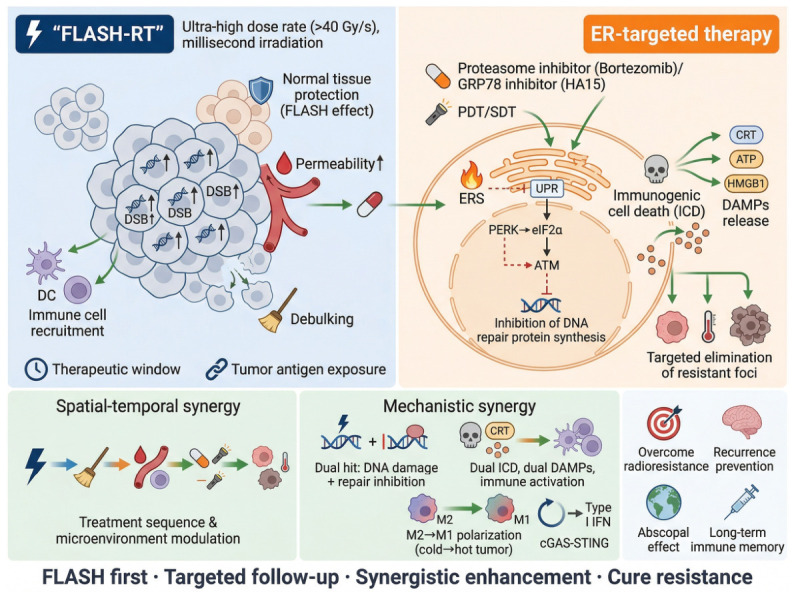
Conceptual macroscopic framework for FLASH-RT combined with ER-targeted radiosensitization. FLASH-RT delivers ultra-high-dose-rate irradiation (>40 Gy/s) within milliseconds to seconds and may widen the normal-tissue therapeutic window through the FLASH effect. ER-targeted therapy can induce ERS through pharmacological approaches, such as proteasome or GRP78 inhibition, or through physical approaches, including photodynamic or sonodynamic strategies. Under sustained stress, the UPR may suppress repair protein synthesis and promote ICD with release of DAMPs such as CRT, ATP, and HMGB1. The proposed interaction between these modalities is conceptual and requires direct validation in GBM models. The figure should therefore be interpreted as a hypothesis map rather than as evidence of established FLASH-specific synergy.

Whether delivered sequentially or in a coordinated manner, the integration of FLASH-RT with ER-targeted therapy should be evaluated according to specific mechanistic and translational criteria: preservation of normal brain function, reliable tumor and subcellular delivery of the sensitizer, compatibility between the millisecond time scale of FLASH delivery and the hours-to-days pharmacology of targeted agents, and direct evidence of improved tumor control in orthotopic and immunocompetent GBM models. These criteria are more appropriate than assuming synergy from conceptual complementarity alone. The proposed macroscopic relationship between FLASH delivery, ER-targeted stress induction, and candidate immune or repair-related endpoints is summarized in Figure 2.

### 5.2. Hypothesized Molecular Intersections That Merit Experimental Testing

**Figure 3:**
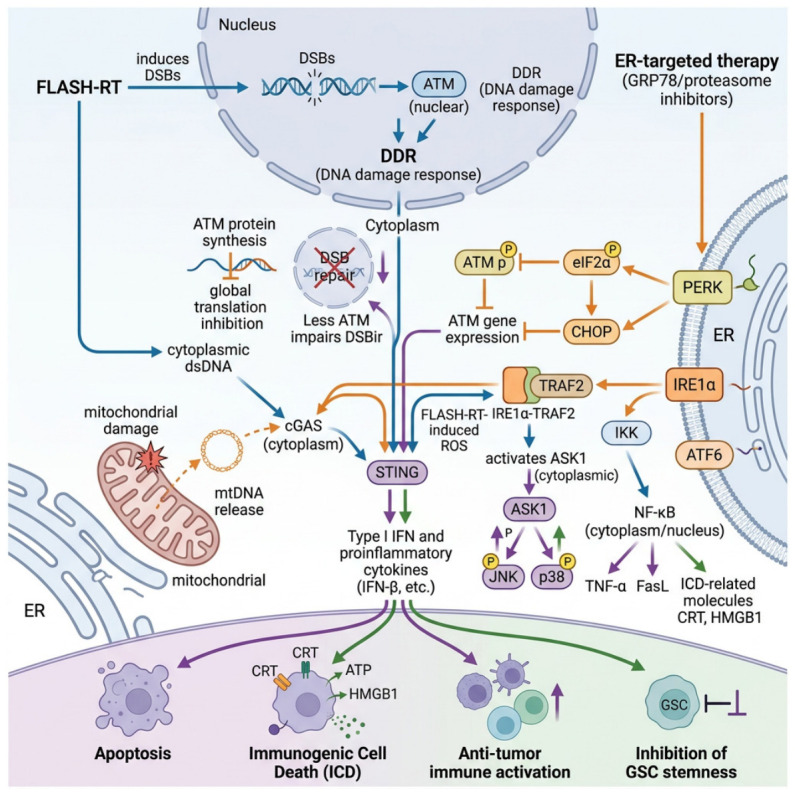
Hypothesized molecular intersections between FLASH-RT and ER-targeted therapy. This conceptual signaling map summarizes possible points of convergence among ERS/UPR signaling, DNA damage responses, inflammatory signaling, and ICD-related immune activation. The PERK-eIF2alpha axis may reduce synthesis of selected repair-associated proteins, potentially limiting repair of radiation-induced DSBs. The IRE1alpha-TRAF2 and cGAS-STING pathways may link ERS, mitochondrial damage, cytoplasmic DNA sensing, type I interferon signaling, and inflammatory outputs. ASK1-JNK/p38 and IKK-NF-kappaB signaling may further contribute to apoptosis or inflammatory modulation. These mechanisms are shown as experimentally testable hypotheses and should not be interpreted as direct evidence that FLASH-RT plus ER targeting overcomes GBM radioresistance.

Because direct GBM studies combining FLASH-RT with ER-targeted interventions are currently unavailable, the pathways discussed below should be interpreted as mechanistic hypotheses derived from ER stress biology, conventional radiotherapy radiosensitization studies, and radiation immunology. They identify experimentally testable nodes of convergence rather than established mechanisms of synergy in GBM. In this framework, FLASH-RT and ER-targeted therapy may intersect at several molecular nodes (Figure 3).

#### 5.2.1. Intersection of DNA Damage and ERS Signaling at the Level of Repair Inhibition: The PERK-ATM/ATR Axis

Radiation-induced DSBs activate DDR kinases such as ATM and ATR, whereas ER-targeted therapy may activate the PERK branch of the UPR. Activation of the PERK/eIF2alpha pathway can suppress global protein translation, thereby reducing the synthesis of selected DNA repair proteins, including ATM, DNA-PKcs, and BRCA1 [34]. In the setting of FLASH-RT, where rapid DNA damage induction may create a high demand for repair machinery, ER-targeted therapy could theoretically constrain the availability of repair proteins and delay damage resolution. This proposed interaction provides a testable rationale for combining DNA damage induction with impaired repair capacity, but it remains to be demonstrated directly in GBM FLASH-RT models.

#### 5.2.2. Intersection of Cell Death and Immune Activation Signals: The IRE1α-TRAF2-JNK/NF-κB and cGAS-STING Pathways

The potential amplification of ICD by ERS, together with immunogenic signals triggered by radiation, may depend on several shared downstream signaling hubs. The following pathways are therefore presented as candidate intersections for experimental testing, not as confirmed FLASH-specific synergy.

MAPK/NF-kappaB pathway. Upon activation, the ERS sensor IRE1alpha can recruit TRAF2 through its cytoplasmic domain, thereby engaging JNK and NF-kappaB signaling. JNK signaling may promote pro-apoptotic mediators, whereas NF-kappaB regulates pro-inflammatory factors and immune modulators. Radiation-induced DNA damage and ROS can likewise activate MAPK family members, including JNK and p38, as well as NF-kappaB signaling. Convergence of these pathways could amplify pro-apoptotic and pro-inflammatory outputs, but the strength and direction of this interaction must be tested in GBM models under FLASH conditions.

cGAS-STING pathway. This pathway represents a key link between DNA damage and innate immune activation. Cytosolic DNA fragments generated after FLASH-RT, whether derived from the nucleus or mitochondria, can be sensed by cGAS, leading to production of cGAMP, activation of STING, and subsequent induction of immune-stimulatory mediators such as type I interferons [17]. Notably, the ER is a critical platform for STING activation and trafficking. ERS may influence STING signaling by altering ER calcium homeostasis or through direct regulatory interactions with STING. In addition, emerging evidence suggests that ERS-related signals may engage in crosstalk with the cGAS-STING pathway through mechanisms that remain incompletely understood. Thus, FLASH-RT may provide the initiating signal in the form of cytosolic DNA, whereas ER-targeted therapy may enhance the cellular context required for STING activation, together promoting robust activation of the cGAS-STING axis and more efficient conversion of physical and biochemical damage into antitumor immune signaling [48].

Taken together, the mechanistic synergy between FLASH-RT and ER-targeted therapy is likely to arise from coordinated interactions among multiple signaling pathways. At the level of DNA repair, PERK-dependent inhibition of DDR protein synthesis may render FLASH-induced DNA damage more persistent. At the level of cell death and immune activation, both modalities converge on major signaling hubs, including MAPK/JNK, NF-κB, and cGAS-STING, thereby generating a coordinated pro-apoptotic and pro-inflammatory network. These interactions provide a plausible mechanistic basis for the observed enhancement of ICD and antitumor immune activation, and support the rationale for further optimization of this combinatorial strategy.

## 6. Other Organelle-Targeted Radiosensitization Strategies as Comparators

**Figure 4:**
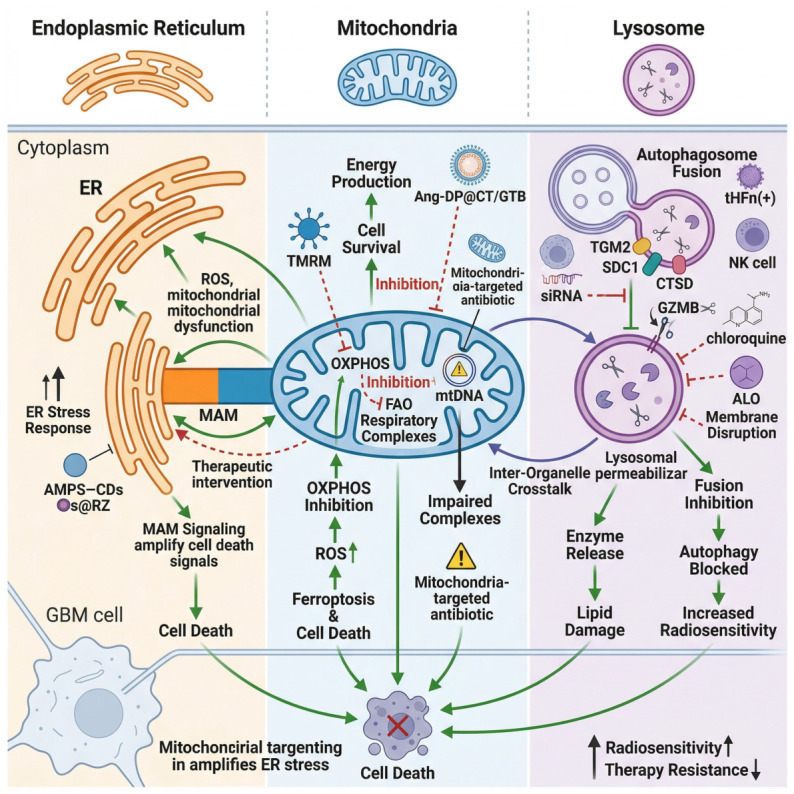
Comparison of organelle-targeted radiosensitization strategies and their evidence boundaries. ER, mitochondrial, and lysosomal approaches may influence distinct but interconnected resistance pathways in GBM. ER targeting may perturb proteostasis and UPR signaling; mitochondrial targeting may disrupt oxidative phosphorylation, ROS handling, and ferroptosis-related processes; lysosomal targeting may alter autophagy, membrane permeabilization, and immune cell interactions. Most supporting evidence for these strategies comes from conventional irradiation or non-FLASH settings. The figure is therefore intended as a comparative conceptual map rather than proof of FLASH-specific synergy.

In addition to the ER, mitochondria and lysosomes represent organelle-targeted radiosensitization strategies supported mainly by conventional-irradiation studies; their relevance to FLASH-RT remains hypothetical (Figure 4). ER targeting is prioritized here as a lead biological hypothesis, not because it has been proven superior, but because it may intersect several resistance layers simultaneously, including proteostasis, DNA repair protein synthesis, immunogenic cell death, and stress adaptation. Mitochondrial, lysosomal, nanoparticle-enabled, and other delivery-based approaches remain important comparators or complementary strategies.

### 6.1. Mitochondrial-Targeted Therapy

GBM cells, particularly GSCs, often exhibit marked mitochondrial metabolic reprogramming, making mitochondria attractive therapeutic targets because of their central roles in energy production and apoptosis regulation [77]. Mitochondrial dysfunction can directly trigger cell death, and the dependence of GBM cells on mitochondrial activity may therefore provide a therapeutic vulnerability [78]. For example, under glucose-deprived conditions, GBM cells become increasingly reliant on mitochondrial OXPHOS for growth. In this setting, antibacterial agents that inhibit mitochondrial translation have been shown to induce mitochondrial dysfunction and ferroptosis [79]. Likewise, interventions targeting mitochondrial metabolism, such as inhibition of fatty acid oxidation (FAO), may attenuate GBM radioresistance and help reverse immune evasion phenotypes [80].

A range of mitochondria-targeted agents and photosensitizers have been developed to enhance the effects of radiotherapy by disrupting OXPHOS, increasing ROS generation, or inducing mitochondrial membrane permeability transition. For example, the mitochondria-targeted photosensitizer tetramethylrhodamine methyl ester (TMRM) can induce mitochondrial damage and irreversible loss of membrane potential in GBM cells during PDT, ultimately leading to cell death [81]. In terms of delivery, nanotechnology-based platforms are widely used to improve mitochondrial targeting and enhance drug accumulation in tumor tissue through functional modification of carrier systems [82].

### 6.2. Lysosomal-Targeted Therapy

As central organelles for cellular degradation and recycling, lysosomes represent another important therapeutic target in GBM. Increased lysosomal membrane permeability can result in the release of hydrolytic enzymes and trigger cell death, making lysosomal disruption a potentially effective antitumor strategy. Various approaches have been developed to target lysosomes, with nanomedicine and PDT showing particular promise. For example, bioengineered protein nanocarriers based on human H-ferritin can respond to mildly acidic environments and dissociate within endosomal compartments, thereby promoting escape of loaded siRNA from lysosomes and enabling targeted RNA interference therapy in GBM [83]. In addition, direct disruption of lysosomal membrane integrity represents another effective strategy. The antimalarial drug mefloquine, for instance, exerts both anti-angiogenic and antitumor effects by impairing lysosomal integrity and function in GBM microvascular endothelial cells [84].

Inhibition of lysosomal function also has profound effects on autophagic flux, and autophagy plays a complex and context-dependent role in GBM radioresistance. Studies have shown that autophagy, particularly the fusion of autophagosomes with lysosomes, is a critical process supporting radioresistance in GBM. Upregulation of cathepsin D has been associated with radioresistance, whereas its inhibition impairs autophagosome–lysosome fusion, reduces autophagic activity, and ultimately enhances radiosensitivity [85]. Similarly, overexpression of transglutaminase 2 (TGM2) and syndecan-1 (SDC1) is associated with poor prognosis and radioresistance in GBM, as these molecules cooperatively regulate autophagosome–lysosome fusion. Knockdown of either SDC1 or TGM2 disrupts this process and increases the radiosensitivity of GBM cells [86].

### 6.3. Inter-Organelle Interactions and Combined Targeting

Mitochondria and the ER are closely connected through mitochondria-associated membranes (MAMs). This structural and functional coupling implies that targeting one organelle may influence the function of the other, thereby generating amplified cellular stress responses. For example, a carbon dot-based nanotherapeutic agent combined with riluzole has been reported not only to target mitochondria, but also to disrupt intracellular membrane systems, including the ER, ultimately leading to nuclear membrane blebbing and cell death [87]. These findings suggest that mitochondria-targeted interventions may transmit stress signals to the ER via MAMs and thereby induce broader stress responses within tumor cells.

Crosstalk also exists between lysosomal dysfunction and ERS. In the setting of radiotherapy or treatment with ERS inducers, selective inhibition of cytoprotective autophagy pathways, particularly autophagosome–lysosome fusion, may represent a promising combinatorial strategy for overcoming GBM treatment resistance [87]. Accordingly, the development of integrated approaches that simultaneously target mitochondria, lysosomes, and related organelle-associated signaling pathways may offer substantial synergistic antitumor effects.

## 7. Challenges in Clinical Translation and Potential Pathways Forward

### 7.1. Clinical Translation Challenges of FLASH-RT Combined with ER-Targeted Therapy

#### 7.1.1. Equipment and Technical Bottlenecks: Implementation Challenges from Electrons to Protons and Photons

Electron-beam FLASH technology is currently among the most rapidly advancing platforms in clinical translation. Prototype clinical devices have achieved UHDR irradiation (≥40 Gy/s) in animal models and demonstrated significant tissue-sparing effects [88]. However, the physical characteristics of electron beams limit their penetration depth, making them generally suitable only for superficial tumors. For deep-seated brain tumors such as glioblastoma, they are unlikely to meet the requirements of whole-brain irradiation or treatment of large target volumes [89]. Proton therapy, by contrast, offers substantial promise for normal brain tissue sparing because of its favorable Bragg peak properties and is therefore considered an attractive platform for achieving the FLASH effect, particularly in central nervous system tumors [90]. Nevertheless, the extremely high cost and maintenance demands of proton FLASH systems, including cyclotrons and synchrotrons, greatly restrict their accessibility and hinder broader research and clinical implementation [90]. In comparison, photon (X-ray) FLASH technology based on existing medical linear accelerator platforms may represent the most practical route for integration into current radiotherapy centers. However, achieving stable UHDR photon output remains technically challenging, requiring extensive modifications to pulse frequency, target materials, and beam control systems to ensure that FLASH dose-rate thresholds can be reached consistently while still satisfying clinical dosimetric requirements such as dose uniformity and conformality [89]. Collectively, these technical limitations remain major barriers to the clinical translation of FLASH-RT for deep-seated brain tumors.

#### 7.1.2. Drug Delivery Barriers: Crossing the BBB and Achieving Subcellular Organelle Targeting

The BBB is essential for maintaining the brain microenvironment, but it also constitutes a major obstacle in the treatment of glioblastoma. It severely restricts the penetration of most therapeutic agents, including both small-molecule inhibitors and biologics, into tumor tissue, thereby limiting intratumoral drug accumulation and substantially reducing therapeutic efficacy [91]. Even when drugs are able to cross the BBB and enter tumor cells, an additional challenge arises for ERS-targeted agents: how to deliver them efficiently and selectively to the ER without degradation, sequestration, or inactivation during intracellular trafficking, particularly by lysosomes or other organelles. Overcoming this problem is critical for improving therapeutic efficacy while minimizing off-target toxicity. Nanodelivery systems have shown considerable promise in this regard. For example, glioblastoma-derived exosomes and other small extracellular vesicles exhibit natural tropism for brain tumors and may therefore serve as carriers capable of crossing the BBB and delivering chemotherapeutic agents such as TMZ to tumor sites [92,93]. However, the design of such nanocarriers requires simultaneous optimization of multiple complex parameters, including BBB penetration efficiency, tumor-targeting specificity, controlled drug release, and in vivo biosafety. As a result, their development remains technically demanding and is still largely confined to the preclinical stage.

#### 7.1.3. Scientific Gaps in Optimizing Combination Therapy Regimens

For combination strategies involving FLASH-RT and ER-targeted therapy, the optimal treatment sequence remains unclear. Different schedules of drug administration and irradiation may produce substantially different biological effects by altering tumor vascular permeability, the immune microenvironment, and the activation status of ERS-related signaling pathways. At present, however, systematic preclinical evidence to guide this critical aspect of treatment design is lacking [94]. Similar uncertainties also apply to dose fractionation. It remains unknown whether FLASH-RT should be delivered as a single high dose or in multiple fractions, and how fraction number and interval should be coordinated with the dosing schedule of ER-targeted agents, such as weekly or every-three-week administration, in order to achieve maximal synergy. Evidence addressing these questions is currently very limited. Some studies suggest that, in glioblastoma, single high-dose irradiation (e.g., 25 Gy) or extreme hypofractionation may be required to induce substantial immune cell infiltration within the TME [94]. In CONV-RT, however, such regimens are constrained by excessive toxicity. FLASH-RT may provide a potential solution, but the optimal way to integrate it with targeted agents remains unresolved. In addition, the absence of robust biomarkers capable of predicting patient sensitivity to this combination strategy, together with the lack of reliable indicators, such as imaging or liquid biopsy markers, for dynamic monitoring of therapeutic efficacy and adverse effects, represents another major obstacle to precision treatment and individualized regimen design. For example, urinary D-asparagine has been reported to be associated with GBM and may serve as a potential diagnostic or monitoring biomarker [95]. Overall, direct preclinical evidence supporting this combination strategy remains insufficient, and systematic validation is urgently needed.

### 7.2. Potential Pathways Forward

#### 7.2.1. Potential Pathway 1: FLASH Platform Development and Clinical Adaptation

(1) Breakthrough Progress in Chinese Photon FLASH Technology

China has made notable advances in the field of photon FLASH technology. Traditionally, the FLASH effect was thought to be primarily associated with charged particles, such as electrons and protons. However, work by the team at the China Academy of Engineering Physics has challenged this assumption by demonstrating that high-energy X-rays may also produce the FLASH effect [96]. This finding opens a new avenue for implementing FLASH radiotherapy using the linear accelerator platforms already widely available in clinical practice. Building on this work, the same team developed a 10 MeV photon FLASH experimental prototype with key performance metrics reaching internationally advanced levels. At a source-to-surface distance of 1 m, the system achieved a dose rate of 80.5 Gy/s, substantially exceeding the widely recognized FLASH threshold of approximately 40 Gy/s [97]. This technological advance not only highlights China’s growing capacity in this field but also points to a potentially more accessible FLASH platform with relatively lower deployment costs and greater compatibility with existing radiotherapy infrastructure. To further clarify the underlying biological basis of the FLASH effect, researchers have also proposed a novel hypothesis centered on differences in “DNA integrity”, providing an additional perspective on how normal tissues may be preferentially spared under UHDR irradiation [98]. According to this concept, UHDR exposure may generate differential DNA damage responses in tumors and normal tissues by influencing the initial burden of DNA damage or the kinetics of its repair. This framework may also offer a theoretical basis for future combination strategies, particularly those involving DNA damage response inhibitors.

(2) Application of the Hybrid UHDR Radiotherapy Strategy

For whole-brain irradiation or treatment of large target volumes such as glioblastoma, the Hybrid UHDR (HUC) strategy may represent a particularly promising clinical approach. The rationale of this strategy is to combine the conformal dose distribution achievable with conventional dose-rate radiotherapy and the normal tissue-sparing potential of UHDR irradiation. In practice, conventional dose-rate volumetric modulated arc therapy (VMAT) is used to cover most of the target volume, whereas UHDR electron beams are applied as a boost to high-risk regions, such as the tumor bed or areas at high risk of recurrence [99]. Studies suggest that, using this hybrid design, approximately 50–69% of the target dose can be delivered under UHDR conditions, thereby maximizing exploitation of the FLASH effect while maintaining adequate target coverage. Preclinical models have provided encouraging evidence supporting the benefits of the HUC approach. Compared with entirely CONV-RT, the HUC regimen has been reported to achieve a brain tissue-sparing effect of approximately 31–32% [100]. This reduction may be particularly important for functionally critical neural structures such as the hippocampus. Indeed, the HUC strategy may substantially reduce radiation exposure to these regions, for example by maintaining the mean hippocampal dose below 6 Gy, which could be highly relevant for preserving cognitive function and improving long-term quality of life [101].

(3) Upgrade Pathways for Existing Radiotherapy Centers

For radiotherapy centers already equipped with widely installed medical linear accelerators, upgrading existing systems to achieve FLASH capability may represent an economically realistic pathway. This approach would rely on targeted modification of several key components, including replacement of the electron gun and accelerating tube with high-power versions to increase pulse frequency to ≥300 Hz, upgrading to a high-speed rotating tungsten target capable of tolerating higher instantaneous power, and incorporating a dedicated UHDR dose monitoring system to ensure accurate dosimetry and treatment safety under ultra-high-dose-rate conditions [48]. Together, these modifications are intended to enable existing systems to generate photon beams that meet FLASH requirements. For newly planned radiotherapy centers or research-oriented hospitals, direct procurement of dedicated domestic X-Flash radiotherapy systems may provide a more straightforward hardware solution for photon FLASH treatment, thereby avoiding the technical complexity, added costs, and possible uncertainties associated with secondary retrofitting of conventional systems [88]. In addition to hardware adaptation, optimization of treatment planning is also critical for realizing the full potential of FLASH-based combination strategies. Studies have suggested that BBB permeability may peak within a defined time window, for example 24–48 h after photon FLASH irradiation [100]. This interval may provide an opportunity for subsequent intravenous administration of nanomedicines or targeted therapeutic agents, thereby facilitating greater drug penetration into the tumor and enabling spatiotemporal coordination between radiotherapy sensitization and systemic treatment. Such an approach may offer a promising framework for combination therapy in GBM.

#### 7.2.2. Potential Pathway 2: Intelligent Nanodelivery Systems to Overcome the BBB and Achieve Subcellular Organelle Targeting

(1) Design and Construction of BBB-Penetrating Nanoliposomes

To overcome the barrier posed by the BBB to drug delivery, the development of nanoliposomes capable of active BBB targeting represents a key strategy. By covalently conjugating ligands that specifically recognize receptors highly expressed on the BBB to the surface of nanoliposomes, receptor-mediated transcytosis can be exploited to actively transport therapeutic agents across the BBB [91]. For example, Angiopep-2, a peptide that targets low-density lipoprotein receptor-related protein 1 (LRP1), is an effective ligand candidate. In terms of payload design and functionalization, nanoliposomes can be engineered to co-encapsulate ERS inducers together with enzymes that modulate the TME. For instance, loading a GRP78 inhibitor such as HA15 may enable direct targeting of the ER in tumor cells, thereby inducing pronounced ERS and promoting apoptosis [102]. At the same time, incorporation of catalase may help decompose the high levels of hydrogen peroxide present in the TME, thereby alleviating oxidative stress, potentially modulating the immunosuppressive microenvironment, and reducing damage to normal tissues [103]. In addition, optimization of the physicochemical properties of nanoliposomes is essential. For example, careful control of particle size within the range of 80–120 nm may help balance circulation time with tumor penetration efficiency. Polyethylene glycol (PEG) modification can further provide a stealth coating, reducing recognition and clearance by the mononuclear phagocyte system and thereby prolonging blood circulation half-life [92].

(2) In Vitro and In Vivo Validation of Targeting and Therapeutic Efficacy

In vitro BBB models and GBM-bearing animal studies support the broader feasibility of ligand-directed and extracellular-vesicle-based delivery systems for improving tumor accumulation and subcellular targeting [85,102,103,104]. However, these data should not be interpreted as proof that HA15 nanoliposomes or other ERS-inducing nanomedicines will synergize with FLASH-RT in GBM. For the present framework, the key translational questions are whether a candidate agent can cross heterogeneous BBB regions, reach infiltrative margins, localize to the intended organelle, and maintain an adequate intratumoral exposure window relative to the time course of radiation-induced biological responses.

#### 7.2.3. Potential Pathway 3: Early-Phase Feasibility Study Design and Combination-Regimen Optimization

(1) Principles for Early-Phase Feasibility Testing

Before a specific clinical trial schema can be justified, the combination of FLASH-RT and ER-targeted therapy should undergo staged feasibility testing. Patient selection, dose constraints, drug choice, and treatment sequence should be derived from platform-specific dosimetry, pharmacokinetic and biodistribution data, and orthotopic GBM safety studies. Rather than prespecifying a single multi-arm Phase I design or fixed drug-administration window, early clinical planning should prioritize safety, normal brain constraints, target engagement, and feasibility of delivery in recurrent or focal re-irradiation settings.

(2) Exploratory Endpoints and Biomarker Framework

To comprehensively assess the efficacy and underlying mechanisms of FLASH radiotherapy combined with ER-targeted therapy, a multidimensional framework of exploratory endpoints and biomarkers should be established. In addition to safety as the primary endpoint, secondary efficacy endpoints could include objective response rate according to RANO criteria, median progression-free survival (mPFS), and tumor metabolic changes assessed by radiomics, which may help quantify treatment response at a macroscopic level [89]. Mechanistic validation and dynamic monitoring are also central to understanding treatment effects. Tumor tissue obtained from biopsy before and after treatment could be analyzed for changes in key ERS pathway proteins, such as GRP78 and CHOP, thereby directly assessing target engagement [102]. Liquid biopsy may provide a complementary noninvasive approach for longitudinal monitoring. Dynamic changes in circulating tumor DNA (ctDNA), particularly MGMT promoter methylation status, could be used to evaluate tumor burden and genomic evolution, and may support response prediction [105]. In parallel, serum levels of exosome-associated GRP78 may serve as a potential biomarker of systemic ERS responses [85]. Furthermore, measurement of circulating DAMPs linked to ICD, such as calreticulin (CRT) and HMGB1, may help estimate the magnitude of treatment-induced antitumor immune activation [103]. Finally, serial neurocognitive assessments, including tests of memory and executive function, would be important for quantifying the potential advantage of FLASH-based treatment in preserving normal brain tissue function [88].

(3) Optimization of Treatment Timing and Radiotherapy Fractionation

Optimization of treatment sequence and fractionation should be guided by measured pharmacokinetics, intratumoral retention, BBB permeability, and biomarkers of UPR activation and immune response. FLASH-RT occurs on a millisecond-to-second time scale, whereas ER-targeted small molecules or nanomedicines act over hours to days; therefore, synergy cannot be assumed from temporal proximity alone. Future studies should test multiple sequencing hypotheses, including pretreatment, concurrent exposure, and post-irradiation administration, while monitoring normal brain toxicity and tumor control in orthotopic models. Only after these prerequisites are met should specific clinical dose levels, intervals, or adaptive fractionation schedules be proposed.

## 8. Conclusions

FLASH radiotherapy combined with ER-targeted radiosensitization is best understood at present as a hypothesis-generating conceptual and translational framework for glioblastoma rather than as an established therapeutic strategy. Available GBM data support the normal-brain-sparing potential of FLASH-RT, but they do not show that FLASH-RT alone reliably overcomes intrinsic GBM radioresistance. Similarly, ER and other organelle-directed interventions provide biologically plausible radiosensitization hypotheses, but direct evidence for FLASH-specific synergy in GBM remains lacking.

Future work should prioritize four questions: whether FLASH-RT expands the safety window for organelle-targeted radiosensitizers in orthotopic, immunocompetent GBM models; which BBB-penetrant agents can achieve reliable subcellular localization in infiltrative margins; which biomarkers best capture UPR activation, ICD, tumor control, and normal brain sparing; and whether early-phase clinical testing should begin in recurrent disease or focal re-irradiation settings. These questions should be addressed before the framework is converted into a specific clinical regimen.

Several translational variables will determine whether this concept is clinically testable. FLASH-RT delivery platforms must achieve reproducible UHDR dosimetry for intracranial targets; ER-targeted agents must demonstrate adequate brain and tumor exposure; combination timing must be matched to drug pharmacology rather than assumed; and safety studies must evaluate neurocognitive, vascular, and immune consequences. These requirements are essential to avoid premature clinical implementation in the absence of adequate mechanistic and safety evidence.

In summary, the strongest rationale for retaining FLASH-RT in this framework is its reproducible preclinical signal of normal-tissue sparing, which may make it useful when additional radiosensitizing stress is layered onto cranial irradiation. The strongest rationale for considering ER targeting is its potential functional intersection with proteostasis, DNA repair protein synthesis, stress adaptation, and immunogenic signaling. The next stage of research should therefore be evidence-stratified, comparative, and mechanism-driven, with ER-, mitochondrial-, lysosomal-, nanoparticle-enabled, and other delivery-based strategies tested side by side rather than assumed to be hierarchically superior.

## Figures and Tables

**Table 1:** Evidence boundaries and hypothesis status for FLASH-RT-based organelle-targeted radiosensitization in glioblastoma.

Core Question	Direct Evidence in GBM	FLASH-Specific Evidence?	Recommended Interpretation
Can FLASH-RT reduce normal brain injury?	Yes; multiple preclinical studies support normal brain sparing.	Yes	Relatively robust preclinical conclusion.
Does FLASH-RT control GBM better than conventional RT?	No clear superiority has been demonstrated.	Yes	State explicitly that superiority is not established.
Is the ER/UPR axis functionally important in GBM?	Yes; moderate evidence supports adaptive UPR involvement in GBM biology and treatment resistance.	No; most evidence is not FLASH-specific.	Candidate vulnerability, not a proven dominant target.
Can ER targeting radiosensitize GBM?	Limited evidence, mostly from conventional RT or non-direct combination settings.	No	Plausible and testable hypothesis.
Has FLASH-RT plus ER targeting been directly validated in GBM?	No direct experimental evidence was identified.	No	Must be framed as hypothesis-generating.
Do mitochondrial or lysosomal strategies support FLASH synergy?	Evidence mainly supports broader organelle-based radiosensitization under conventional RT.	No	Important comparators or complements, not established FLASH-specific synergy.

## Data Availability

No new data were created or analyzed in this study. Data sharing is not applicable to this article.

## Abbreviations

The following abbreviations are used in this manuscript:

GBM	glioblastoma
RT	radiation therapy
FLASH-RT	FLASH radiation therapy
GSCs	glioma stem cells
TME	tumor microenvironment
ER	endoplasmic reticulum
ERS	endoplasmic reticulum stress
UPR	unfolded protein response
DDR	DNA damage response
DSBs	DNA double-strand breaks
NHEJ	non-homologous end joining
HR	homologous recombination
ICD	immunogenic cell death
ROS	reactive oxygen species
BBB	blood–brain barrier
